# Retraction Note: Long non-coding RNA LINC00525 regulates the proliferation and epithelial to mesenchymal transition of human glioma cells by sponging miR-338-3p

**DOI:** 10.1186/s13568-022-01498-4

**Published:** 2022-12-21

**Authors:** Yilv Wan, Feng Liang, Minjun Wei, Ying Liu

**Affiliations:** 1 Deparment of Neurosurgery, The First Affiliated Hospital of Nanchang University, Nanchang, 330006 Jiangxi China; 2 Deparment of Stomatology, The First Affiliated Hospital of Nanchang University, Nanchang, 330006 Jiangxi China; 3 Deparment of Nephrology, Jiangxi Cancer Hospital, Nanchang, 330006 Jiangxi China

**Retraction: AMB Expr (2020) 10:156** 10.1186/s13568-020-01094-4

The Editor-in-Chief has retracted this article. After publication, concerns were raised regarding image overlap within the article, as well as with previously published articles by other authors. Specifically:

- The wound healing images in Fig. 3 representing U118 si-NC and si-LINC00525 groups at 0h appear to overlap.
- The cell images in Fig. 4 appear highly similar to those in Figs. 3B and 5C in [1] and in Figs. 2F, 2G, 4G and 4H in [2].

The Editor-in-Chief therefore no longer has confidence in the presented data.

Author Minjun Wei has stated on behalf of all co-authors that they agree to this retraction.
